# COVID-19: The Patients' Perceived Impact on Dental Care

**DOI:** 10.1055/s-0041-1734470

**Published:** 2021-11-16

**Authors:** Alessandra Amato, Alfredo Iandolo, Giuseppe Scelza, Francesca Spirito, Stefano Martina

**Affiliations:** 1 Department of Medicine, Surgery and Dentistry, Scuola Medica Salernitana University of Salerno, Baronissi, Italy

**Keywords:** aesthetics, COVID-19, dental practice, diet, survey

## Abstract

**Objectives** The present study aims to investigate the effect of the COVID-19 pandemic on people's mental and physical balance, oral hygiene habits, type of diet, perceived safety of returning to the dentist, and aesthetics with the use of masks.

**Materials and Methods** An online questionnaire was submitted to the Italian population between December 2020 and January 2021. It was sent via online platforms and included 21 questions.

**Statistical Analysis** Differences in rates were calculated by using the Chi-square test. The level of significance was set at *p* <0.05.

**Results** A total of 1,008 individuals completed the questionnaire. About 72% of participants were not concerned about returning to the dentist. Approximately 45% of the individuals intensified their oral hygiene and preventive rules. About 38% of participants increased their carbohydrate intake, while 28% increased their fat consumption. Furthermore, 75% of the participants felt that the mask did not diminish the beauty of their smile.

**Conclusions** Most participants felt comfortable returning to the dentist but only for more urgent treatment. However, most people reported that they had not stepped up their home oral hygiene measures. The biggest changes in the population's eating habits involved increased carbohydrate and fat consumption. Finally, most participants responded that mask use did not compromise their aesthetics.

## Introduction

In December 2019, a new viral infection broke out in Wuhan, a city in central China. 1 In January 2020, the etiologic agent was identified and named Severe Acute Respiratory Syndrome Corona Virus 2 (SARS-CoV-2). 2 The World Health Organization (WHO) recognizes the disease as COVID-19, an acronym derived from the combination of the terms “Co-rona Vi-rus D-isease” and the year of identification 2019. 3 The rapid spread of the virus across countries worldwide compels the WHO to declare global pandemic status on March 11, 2020. 4

Human-to-human transmission of SARS-CoV-2 occurs through close contact and exposure to respiratory droplets, with asymptomatic patients representing a discrete source of infection. 5

In March 2020, because of the enormous risk of transmission of the infection, the Italian government and other countries adopted restrictive quarantine and lockdown measures to limit the spread of the disease and deaths. On May 4, 2020, at the end of the quarantine period, phase 2 of the health emergency begins with the gradual reopening of production and commercial activities and the obligation to respect the social distance and the use of masks. 6 As of June 3, 2020, inter-regional movement was unblocked, and during the summer months, virus transmission was under control, although there were a few new cases. 7 In the months between autumn and winter, the increase in COVID cases—recognized as a “second wave”—imposed new restrictive measures by the Italian Government on November 3, 2020. Through 21 parameters inherent to monitoring capacity, degree of diagnostic, investigation and contact tracing capacity, and characteristics of transmission dynamics and resilience of health services, Italian regions were assigned to a color by using the traffic light method. Each Italian region according to high, medium, or low risk level assumed the color red, orange, or yellow, respectively. 8

In addition to the disastrous effect on the economic balance, the restrictive measures have had a negative impact on people's psychological status. Some recent studies have observed negative effects on mental health due to factors such as loneliness, stress, anxiety, fear of contracting the virus, depression, and sleep disorders. 9 10 11 Some studies have analyzed the impact of the pandemic period on lifestyle and eating habits. 12 13 The increase in sedentariness, the reduction of sports activities, and the condition of continuous psychological stress induced a change in daily diet, recording a higher intake of sweet and carbohydrate-rich foods. 14 These foods seem to have a positive effect on the production of serotonin and therefore can provide relief for stress and anxiety. 15 However, some studies have also observed an improvement in dietary patterns. In fact, increased consumption of fruits and vegetables and a reduction in alcoholic beverages emerged during the pandemic. 16 17

The pandemic period also affected the perceived risk of infection within dental practices. In fact, Martina et al, through a questionnaire sent to a sample of 1,500 Italian people, observed that 45% of patients considered the dental practice a place at high risk of contagion and 43% considered going to the dentist a risk. 18 Similarly, several studies have highlighted dentists' fear of being infected within their office or infecting people in contact with them. 19 20 21 22

Likewise, a study performed in Spain found that dental practices and hospitals were considered places at increased risk of infection, although 91.6% of patients were not concerned about contracting the virus in the dental practice. 23

The aim of the study was to evaluate if COVID-19 affect the psychophysical balance of people, investigating the oral hygiene habits and the type of diet during the pandemic, how the aesthetic was affected by the use of face mask, and the perception of safety of returning to dentist.

## Materials and Methods

This study was conducted by a survey questionnaire sent to the entire population in Italy via Internet in a period between December 2020 and January 2021. The home page provided information on the scope and purpose of the study.

The survey was created with SurveyMonkey and were transmitted by WhatsApp, Facebook, or e-mail. There were no incentives for participation.

The questionnaire consisted of 21 items that evaluated demographic information; social information; frequency of visit to the dentist before pandemic and possible diagnosis of any disease such as caries, periodontal disease and oral lesions; and changes in home oral hygiene habits. In addition, the questionnaire investigated the influence that the pandemic period had on eating habits, measuring the increase or decrease in consumption of carbohydrates, proteins, fats, sweets, fruits, vegetables, and alcoholic beverages.

Finally, two questions analyzed whether the use of the mask influenced the perception of their smile and facial aesthetics.

The results were transcribed by using an ordinary scale. Participants also had the opportunity to add free text at the end of the questionnaire. We calculated the average time of 5 minutes to complete it.

## Statistical Analysis

The categorical variables were expressed as frequency. Differences in rates were calculated using the Chi-square test. The significance level was set below 0.05. The statistical program used was the Statistical Package for Social Sciences (SPSS) for Windows, version 12.0.

## Results

A total of 1,008 (413 males and 595 females) individuals completed the survey. Information on the age of the individuals showed that most of the participants were included in the age ranges of 18 to 29 years old (388; 38.5%) and 30 to 49 years old (319; 31.6%). About 890 (88.3%) individuals lived at home with family, while the remainder lived alone (94; 9.3%) or at home with friends (24; 2.4%). The Table 1 showed general data of the participants.

**Table 1:** Characteristics of the responders

	Frequency (percentage)					
Age	18–29 388 (38.5%)	30–49 319 (31.6%)		50–69 277 (27.5%)		70–79 24 (2.4%)
Gender	Males 413 (41.0%)			Females 595 (59.0%)		
Residence	Within the family 890 (88.3%)		Alone 94 (9.3%)		At home with friends 24 (2.4%)	

A total of 780 (77.4%) individuals said that they were not afraid when going to the dentist ( Table 2 ). Of these, 56.2% (438/780) were women ( *p* = 0.001). Responses on the frequency of dental visits in the pre-pandemic period showed that most participants visited their dentist once every 6 months (424; 42.1%) or once a year (397; 39.4%). Furthermore, during visits before the pandemic, 313 (31.1%) patients showed the presence of caries, 168 (16.7%) had been diagnosed with periodontal disease (gingivitis or periodontitis), and 141 (14%) had altered oral conditions (mouth ulcers, white lesions of the tongue or cheeks, burning of the mouth, and pink lesion). In addition, 198 (19.4%) people had fixed or removable prostheses in their mouths.

**Table 2:** Frequencies and percentages of responses to questions about perceived risk in returning to the dentist, oral condition, and confidence in returning to the dentist

	Frequency (percentage)				
Fear of the dentist	Yes 228 (22.6%)			No 780 (77.4%)	
Frequency of dental visits in the pre-pandemic period	Never 81 (8.0%)	Once a week 20 (2.0%)	Once a month 86 (8.5%)	Once every 6 mo 424 (42.1%)	Once a year 397 (39.4%)
Diagnosis of caries before the pandemic	Yes 313 (31.1%)			No 695 (68.9%)	
Diagnosis of periodontal disease before the pandemic	Yes 168 (16.7%)			No 840 (83.3%)	
Diagnosis of altered oral conditions before the pandemic	Yes 141 (14.0%)			No 867 (86.0%)	
Presence of fixed or removable prosthesis in the mouth	Yes 196 (19.4%)			No 812 (80.6%)	
Intensification of oral hygiene and preventive rules	Yes 451 (44.7%)			No 557 (55.3%)	
Confidence in returning to the dentist	Yes 724 (71.8%)			No 284 (28.2%)	
Think of carrying out only the most urgent therapies	Yes 718 (71.2%)			No 290 (28.8%)	

Overall 451 (44.7%) participants intensified their oral hygiene and preventive rules (mouthwash, flossing, bottle brushing, and feeding) during the pandemic period, especially women; in fact, 63.4% (286/451) of them were women ( *p* = 0.011).

Since the end of the quarantine, concern about returning to the dentist involved 284 (28.2%) participants. Notably, among them, 66.9% (190/284) were women ( *p* = 0.001); however, 71.2% (718/1,008) of participants expressed a willingness to perform only the most urgent therapies. Of these, women accounted for 62.3% (447/718; *p* = 0.001).

The survey analyzed whether the pandemic period had changed eating habits, and 380 (37.7%) individuals increased their carbohydrate consumption and 282 (28%) consumed more fat and 229 (22.7%) increased protein. Interestingly, fruit intake increased for 405 (40.2%), alcohol or drink consumption decreased for 286 (28.4%), and remaining unchanged for 564 (56%) participants. Regarding the consumption of sweets and snacks, there was an increase for 394 (39.1%) participants, mostly women; in fact, 66.8% (263/394) of them were women ( *p* = 0.000).

The survey explored the opinions about the influence of the mask on aesthetics. For 760 (75.4%) participants, the mask did not generate aesthetic discomfort, while 246 (24.4%) believed that wearing a mask diminished the beauty of their smile; specifically, 76.8% (189/246) of them were females ( *p* = 0.001). In addition, 875 (87%) participants responded that the smile hidden by the mask did not make them more confident about attraction. The data are reported in Table 3 .

**Table 3:** Frequencies and percentages of responses to questions about changes in eating habits and aesthetics

	Frequency (percentage)			
Carbohydrate intake during the pandemic	Decreased 108 (10.7%)	Increased 380 (37.7%)		Unchanged 520 (51.6%)
Protein intake during the pandemic	Decreased 75 (7.4%)	Increased 229 (22.7%)		Unchanged 704 (69.8%)
Fat intake during the pandemic	Decreased 184 (18.3%)	Increased 282 (28.0%)		Unchanged 542 (53.8%)
Intake of fruits and vegetables during the pandemic	Decreased 79 (7.8%)	Increased 405 (40.2%)		Unchanged 524 (52.0%)
Alcoholic beverage intake during the pandemic	Decreased 286 (28.4%)	Increased 158 (15.7%)		Unchanged 564 (56.0%)
Intake of sweets and snacks during the pandemic	Decreased 190 (18.8%)	Increased 394 (39.1%)		Unchanged 424 (42.1%)
Decreased smile beauty from mask use	Yes 246 (24.4%)		No 760 (75.4%)	
Smile hidden by mask makes you feel more confident	Yes 131 (13.0%)		No 875 (86.8%)	

## Discussion

The aim of the study was to evaluate if COVID-19 affect the psychophysical balance of people, investigating the oral hygiene habits and the type of diet during the pandemic, how the aesthetic was affected by the use of facemask, and the perception of safety of returning to dentist.

The sample was an acceptable representation of the different age ranges and different work occupations.

In total, 28.2% (284/1,008) of participants expressed concern about returning to the dentist. These values are lower than the results of a previous study conducted at the end of the quarantine period, in which 45.6% of participants felt it was risky to return to the dentist. 18

The results showed that of the participants who expressed concern about returning to the dentist, 66.9% (190/284) were women ( *p* = 0.001). This is in agreement with Martina 18 and Cotrin, 24 who reported that women had more anxiety and apprehension than men when returning to the dentist.

About 71.2% (718/1008) of participants responded that they would only visit a dentist for emergencies. Notably, 62.3% (447/718) of them were females ( *p* = 0.001). The association with women is consistent with a recent study by Peloso et al, who had observed that men were more inclined to go to the dentist than women who would go only for emergencies. 25

More than half of the participants (55.3%; 557) did not intensify oral hygiene and preventive rules (mouthwash, flossing, bottle brushing, and feeding) during the pandemic period showing less interest in oral health. Similarly, Pinzan-Vercelino et al noted that individuals had a lower frequency of tooth brushing and this was closely associated with an increased prevalence of halitosis. 26

Since the beginning of the pandemic period, there has been a change in eating habits. This has been due to various factors in particular stress and anxiety, greater sedentariness given by smart working, regulations that have reduced people's movements, and changes in the availability of food during the day. 27 28 Therefore, 37.7% (380/1,008) of participants said that they increased their carbohydrate intake. This increase could result from the prevalence of homemade recipes that primarily included foods such as pizza and bread. 14 Moreover, 28% (282) of participants reported increased fat consumption, and increased intake of sweets and snacks was reported in 394 (39.1%) individuals, particularly in women who accounted for 66.8% (263/394) of them ( *p* = 0.000). Di Renzo et al, by means of a survey addressed to approximately 3,500 Italians, observed a clear change in eating habits and an increase in the consumption of “junk food” and sweets. 14 The “junk food” consists of foods rich in energy but poor in essential and healthy nutrients. 29 Further studies observed that the uncontrolled and excessive consumption of this category of food predisposes to the development of chronic pathologies, such as cardiovascular diseases and obesity. 30 31 Moreover, it should be considered that a diet rich in sweets and snacks can increase the risk of dental caries. 32 Therefore, it would be important to give people advice to reduce both the quantity and frequency of sugar intake. Surprisingly, 40.2% (405) of the participants consumed more fruits and vegetables. These results are in agreement with other recent studies that have emphasized increased intake of fresh produce during the quarantine period. 33 34 Likely, the increased consumption of fruits and vegetables was a function of home cooking and WHO awareness of the importance of eating nutritious foods. 35 Fruits and vegetables are foods rich in vitamins and minerals, the lack of which increases the risk of obesity and immune system abnormalities by affecting the response against pathogens. 14

The consumption of alcoholic beverages showed a decrease in 286 (28.4%) participants being in agreement with some studies reporting an important decrease in alcohol intake in the examined population. 34 36 One reason that could explain the decrease in alcohol consumption may be the reduction of social occasions and events, especially in the younger category. 35

The mask, an essential device to reduce the risk of infection of the virus, covers the mouth and perioral area making it more difficult to capture emotional facial expressions such as fear, surprise, sadness, and happiness. This condition can decrease or worsen social communication. 26 Furthermore, 246 (24.4%) participants believed that wearing a mask diminished the beauty of their smile; specifically, 76.8% (189/246) of them were females ( *p* = 0.001). The result and the association with women are confirmed and can also be explained by a study concluded that women really missed looking at people's smile because of its importance in social relationships. 37

The lower half of the face is an essential aspect of attractiveness, and the use of the mask may influence the assessment of beauty parameters. 38 However, 875 (87%) survey participants responded that the smile hidden by the mask did not make them more confident about attraction but was irrelevant. This may stem from a discrete awareness of one's own beauty and attractiveness. In this regard, Patel et al showed that individuals, who without the mask were defined as medium and high attractiveness, after wearing the mask did not differ and did not further increase their attractiveness. 38

This study presents some limitations: it is a survey-based study and thus information is self-reported. However, it also presents some strengths, such as the high number of participants, the good representation of the population, and its depiction of the situation of Italian dental patients.

## Conclusions

This survey-based study investigated the risk perception of Italian people toward attending dental practices and concerning oral hygiene and dietary habits during the COVID-19 pandemic.

Most of the participants felt comfortable returning to the dentist, but only for therapies that are more urgent. Nevertheless, most people declared that they did not intensify their home oral hygiene measures.

There were changes in the eating habits of the population, in particular an increase in the consumption of carbohydrates, fats, and snacks, but the intake of fruit and vegetables also increased.

Finally, most of the participants answered that the use of the mask did not compromise their aesthetics.
